# Rebuilding trust in just culture

**DOI:** 10.1097/01.NUMA.0000891456.44611.10

**Published:** 2022-10-27

**Authors:** Linda Paradiso

**Affiliations:** **Linda Paradiso** is an assistant professor at CUNY School of Professional Studies in New York, N.Y.

## Abstract

Even before RaDonda Vaught was found guilty of criminally negligent homicide, nurses were fearful of being held individually accountable for systemic errors. Leaders are now faced with repairing the loss of faith in just culture. This article provides an understanding of the erosion of just culture and interventions needed to improve trust.

**Figure:**
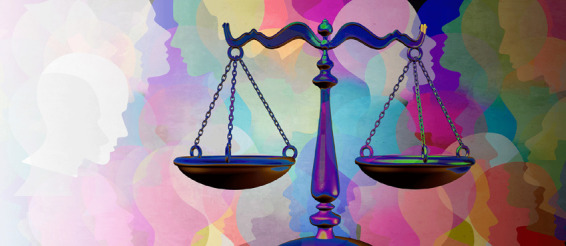
No caption available.

The landmark report in 2000, *To Err is Human: Building a Safer Health System*, brought awareness to why medical errors happen and proposed recommendations for building a voluntary reporting system to improve patient safety.^1^ Designing a safer system requires open and honest communication from direct care workers to leaders and executives. Due to their proximity, expertise, advocacy, and ethical code of conduct, nurses are in a prime position to communicate to leaders the barriers preventing safe patient care. Integral to this voluntary contribution of information regarding near-misses and mistakes is trust that what's reported won't be held against those who report. Healthcare organizations have been working diligently at developing and sustaining just culture environments for many years, and nurses have a deep desire to improve the lives of others and want to care for people in an environment that supports their practice. But they worry about the response when they report.

The intense, hectic, and ever-changing environment nurses work in can drive the erosion of trust. Multitasking, floating to different units, caring for people with unfamiliar diseases, insufficient staffing, and creating work-arounds are experiences that can give rise to the need to improvise, drift, and deviate from policy and standards of practice. Lack of trust is also propelled by nurses' experiences when a mistake is made.

On December 24, 2017, RaDonda Vaught, RN, was challenged with a day that many nurses would describe as typical. She reported to work and was assigned as the “help-all nurse” floating from unit to unit, and she agreed to orient a new nurse. When helping in the radiology unit, she distractedly administered the incorrect medication to Charlene Murphey, who died as a result of her mistake.^2^ Ms. Vaught didn't intend to harm Ms. Murphey and wasn't even aware of her mistake until she heard a cardiac arrest code called. Following this tragic human error, she self-reported what occurred, owning her mistake and taking responsibility. Her actions demonstrated trust in her organization and were aligned with those behaviors expected in a culture of safety.

The events that occurred next brought many nurses' nightmares to life. A cascade of systems issues and deficiencies were identified in a report filed by the Centers for Medicare and Medicaid Services, yet Ms. Vaught was held individually accountable for systemic issues in the hospital where she worked.^2^ She was fired from her job; lost her nursing license; and was arrested, prosecuted, and found guilty of criminally negligent homicide and abuse of an impaired adult.^3^ Her sentence, 3 years of probation, was considered lenient and spared Ms. Vaught from prison. What's the underlying message sent to nurses about trust and just culture?

The Agency for Healthcare Research and Quality (AHRQ) Surveys on Patient Safety Culture (SOPS) report results about healthcare workers' perceptions regarding nonpunitive response to error. The first survey conducted in 2004 had three questions about nonpunitive response to error that have been included in each survey since its inception.^4^ The SOPS Hospital Survey 1.0: 2021 User Database Report included 320 hospitals and 191,977 respondents. The results from these three questions found that more than 45% of healthcare workers perceive that their mistakes are held against them; 49% perceive that if they report an event, they're written up instead of the problem; and more than 59% worry that their mistakes are kept in their personnel file. Nurses represented the largest percentage of respondents with 65,193 completing the survey.^5^ SOPS results reported by AHRQ from 2011 to 2021 reflect small gains, which are encouraging, but all surveys were conducted prior to the criminal prosecution of RaDonda Vaught.^5^-^10^ (See Figure 1.)

**Figure 1:**
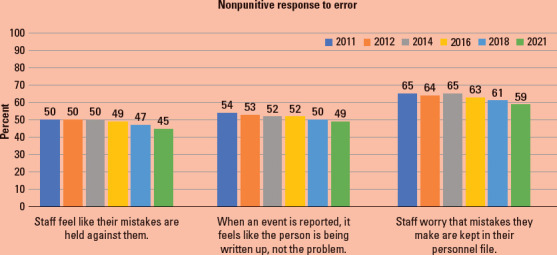
SOPS results reported by AHRQ from 2011 to 2021

In 2017, The Joint Commission (TJC) published Sentinel Event Alert #57, outlining the importance of a nonpunitive response to error in order to create a reliable environment of learning and safe care.^11^ Ms. Vaught knew that there would be changes, but the impact to her, personally, wasn't in her sights. She, like others, believed that when clinicians report openly and honestly, administrators respond with improvements. In learning organizations when this *healthcare learning circle* is complete, future recipients of care can be better protected from the same or similar mistakes.

## Erosion of trust

Despite healthcare organizations' efforts to create and cultivate a just culture, many factors have incrementally undermined healthcare professionals' trust that they'll have the support of their employers should they make a mistake when caring for a patient. Even before RaDonda Vaught's conviction, nurses were fearful of being held individually accountable for systemic errors. Leaders are now faced with repairing the loss of faith in just culture. This article provides an understanding of the erosion of just culture and interventions needed to improve trust.

### Just culture and organizational response to error

In the literature, the definition of just culture is inconsistent, but it's most commonly defined as *the environment in which organizations are accountable for the systems they design and employees are accountable for the choices they make within those systems*.^12^ Within this just environment are pillars of nonpunitive and fair error investigation and the psychologically safe environment in which employees are free to speak up without fear of discipline, retaliation, or retribution.^12^ In healthcare, organizations most often determine the response to error based upon the harm that has occurred. Therefore, a near-miss event doesn't hold the same response weight as a patient death. Ideally, incidents should be analyzed for a root cause and all types of errors, including near-misses, should hold the same learning potential. This is why, when caregivers raise awareness of potential safety issues or impediments to the provision of care, they should be praised and the report should be taken as seriously as if a significant incident occurred. When organizations allow team members to learn from reporting, a better safety culture emerges. When impartial standards are used to review events, the process is destigmatized. When the reporting of an error or near-miss is celebrated, it shapes perception of patient safety culture. This open, bidirectional communication fosters trust between caregivers and leaders with expectations that fair treatment and investigation will occur following an error.^13^

### The incident investigation process

Root cause analysis (RCA), as part of the incident investigation process, is widely used in healthcare. As identified by the AHRQ, it's an error analysis tool used to identify systems issues that lead to unwanted events, and it avoids focusing on individual mistakes.^14^ The RCA goal is to identify and eliminate the contributing factor from a mistake and reduce the likelihood that another event can occur. TJC, which accredits more than 22,000 healthcare organizations and programs throughout the country, has mandated the use of RCA investigations since 1995 to analyze serious incidents in an effort to create an atmosphere of change and improve patient safety.^15^ Kellogg and colleagues noted that solutions identified during RCAs rarely included robust systemic correction or redesign of a process, which are more likely to result in sustained change. Most often, solutions focused on attempts to fix individuals. This study found that the most common outcomes for improvement following an RCA were training and policy reinforcement.^16^ Researchers haven't rigorously studied the RCA process and its effectiveness, and the lack of improvement in medical error may be due to this method being unsuccessful at sustaining change.^16^

### How investigations victimize

Robertson and Long state that “it is a myth that mistakes are rare,” and the stress that's created by promoting perfectionism is victimizing to clinicians.^17^ Analyzing a decision or action from every angle after a poor outcome can include the assumption that the individual's decision-making leading up to the outcome was flawed, when in fact it may not have been.^18^ RCA solutions that identify systems issues need to include redesign and system/organizational accountability. In reality, investigation outcomes most often lead to monitoring and retraining the individual. Retraining should only be required when there's clear evidence that a lack of knowledge contributed to the event. Reviewing policy and procedure or retraining an individual in a task they've previously demonstrated competence in can be perceived as punitive. These types of solutions victimize, don't result in sustained change, and fail to remove hazards that may result in future injury.

Such investigatory outcomes not only affect the culture of safety but can affect the nurse's personal perceptions of trust in leaders, feelings of value to an organization, and overall well-being. It's estimated that 50% of clinicians become a second victim, defined as “a healthcare professional who experiences difficulties to cope with their emotions after a patient safety incident, medical error, or adverse event.”^17^ Participants in a study conducted by Ullström and colleagues noted that a lack of organizational support, or confusing and misdirected support, made processing a significant event more difficult and traumatizing.^19^ Symptoms of second victimization include anxiety, guilt, insomnia, anger, depression, and suicidal ideation.^17^

### Blame culture

The consistent findings from the AHRQ SOPS question, *mistakes are held against me*, confirm that workers worry about individual blame culture following medical error.^5^-^10^ For example, in 2015 following the death of a child, a bill named “Samuel's Law” was proposed in South Carolina mandating the revocation of a nurse's license for misreading a medication order.^20^ The most widely known criminal prosecution of an individual nurse is the previously mentioned RaDonda Vaught case. She was convicted despite evidence that the medical center had significant systemic and departmental deficiencies, neglected to report a fatal incident, and failed to follow its own policies and procedures.^2^ An outcome of this blame approach is that an individual's efforts to avoid personal vulnerability can drive the escalation of safety issues underground.

Okpala defines individual blame logic as the assumption that people are neglectful or don't pay attention to the tasks they're charged to carry out. The author further identifies a related blame culture approach as the monitoring of individuals to enforce strict adherence to policy and procedure.^21^ Although often viewed as education, this can be interpreted as punitive if the clinician was using a variation in order to complete a task. Variation in practice exists because of the imperfect systems in which nurses work. These variations are typically overlooked, lauded as autonomy, and give an illusion that systems are working effectively until a situation or event creates the awareness of risk.^13^

Sidney Dekker opines that death in a place of healing isn't considered a natural death; therefore, a cause and ultimately a person must be responsible.^18^ It's easier to see death emanating from a single action than the alignment of multiple “holes” in a system that attributed to the outcome. Dekker further states that it's easier to allow one individual to carry the blame of many as that one person can shoulder all the pressure, be released, and drop out of sight.^18^

### Criminal prosecution for unintentional human error

Mistakes and crimes aren't alike as mistakes lack *mens rea* or criminal intent.^22^ Criminal prosecution for medical mistakes, though rare, is rising and may reflect a changed view of society's perceptions that there's an achievable expectation of perfection when providing care. TJC, as early as 1996, recognized that human error is inevitable and has methodically provided leadership alerts, guidance, and standards to reduce the likelihood of and promote learning from human error.^11^,^15^,^23^,^24^ Several factors can contribute to the increase in criminal prosecution. In 2009, Alan Merry identified that the goals of compensation, accountability, and retribution are often the result of the actual consequence of the error—for example, death—rather than the moral culpability involved.^25^ Discounting that accidents or mistakes have an innocent origin has increased, possibly because societal culture has encouraged the idea that injury and blame are related. If an injury occurs, there's accountability to be found through monetary compensation, and the law doesn't recognize compensation through potential harm, only actual harm.^26^ Merry also shares that most cases are the result of violation or a deviance from practice rather than an actual error.^25^ Risky decisions made by practitioners, rather than reckless and intentional harm, are far more prevalent, and it's easier to find instances “worthy” of prosecution.

Further, the culture of safety may be impacting the legal system. Research is vitally needed to determine if the *tolerance* of systemic failure has contributed to an explosion for the need to hold *someone* accountable. This tolerance should be considered when reflecting on the trial of RaDonda Vaught. The lack of presence and support by her employer, Vanderbilt Medical Center, was highly visible.^3^ The organization's representatives didn't participate in her defense at the trial to explain *their* system flaws and *their* culture of safety to jurors, and the changes made to *their* system to prevent similar errors from occurring in the future. TJC holds the executive team responsible to ensure a culture of safety, but the judicial system allows for the prosecution of individuals who make mistakes without intent to harm.^11^,^25^

## Rebuilding trust

In light of the many factors that have contributed to the erosion of trust in just culture in recent years, what can organizations do to rebuild this trust? Here are eight interventions you can implement now to start the process:

### 1. Listen to the surveys

The AHRQ has led the nation to improve safety in healthcare since 2001.^4^ The goal of SOPS is to be a useful tool for measuring organizational conditions that can lead to adverse events and patient harm. The surveys measure the culture of the organization's patient safety and safety practices. The surveys are free, valid, and reliable instruments that are easy to administer and can be useful to compare organizations internally and externally.

### 2. Promote teamwork

In 2021, 87% of respondents to AHRQ SOPS reported that people support each other on their unit and, when work needs to be done, they support each other to get the work done.^5^ The survey measures teamwork within and across units. Building on the trust among team members can help an organization to achieve improvements in the safety culture throughout the facility, and nurse leaders should foster this trust.

### 3. Change the systems

Nurses need to work in systems that can protect them against mistakes. Stringent efforts to objectively analyze each event must include direct caregivers so that subtle risks are identified and meaningful improvements are developed. Common system changes to consider are: creating decision-driven policies, realistically standardizing processes and procedures, creating redundancy in all high-risk and high error-prone situations, performing frequent equipment maintenance, correcting education and competency deficiencies without judgment, ensuring adequate staffing, and most important improving communication among team members through simulation activities focused on flattening hierarchical relationships and promoting escalation of concerns to leaders without fear of feeling inadequate.^27^

The role of adequate staffing in systemic events can't be minimized or ignored. Shortages of clinical nurses impact every patient's quality of care and unfinished nursing care related to time constraints.^28^ A study conducted by Brooks Carthon and colleagues in 2020 validated previous studies and found that “higher levels of nurse engagement and more favorable nurse-to-patient staffing ratios were consistently associated with positive ratings of patient safety.”^29^ Careful consideration should be taken to match the method of nursing care delivery to the situation.^30^ For example, employ a functional nursing model when the acuity and number of patients outnumber nurses assigned. Failing to anticipate staffing needs or provide an adequate number of nurses are most often the reasons for policy deviance and can force nurses to make risky decisions and increase the likelihood of human error.

### 4. Investigate objectively

The use of an objective algorithm in determining the type of behavioral choices made during the occurrence ensures that RCA outcomes are clear, fair, and focused. Broder and colleagues report that the use of an algorithm can lead to a standardized investigation where outcome bias can be reduced and fairness is transparent to others.^31^ Including stakeholders, such as injured parties or their representatives, in the root cause process can create humbling and deep learning for all, demonstrate organizational and individual accountability, and repair moral harm.^32^

Investigations should also incorporate support for caregivers to reduce second victimization. Johns Hopkins' *Caring for the Caregiver: The RISE* (Resilience in Stressful Events) Program and the University of Missouri's forYOU Team are two caregiver support programs that encourage their duplication in other facilities.^33^,^34^

### 5. Educate judiciously

The use of reflective learning can be instrumental in the learning process. Hindsight has a clear vision whereas the moment a decision is made often doesn't. Reflective learning not only improves future patient safety but can assist in one's emotional coping following an incident. Provide education and retraining to an individual only if there's a knowledge deficit identified to avoid being perceived as punitive. Frequently it isn't a lack of understanding but actions that deviate and lead to error when policy and procedure can't be carried out as planned.

### 6. Avoid snap judgment

After an event, allow the investigatory process to take its course. No two incidents are alike. Influencing or infecting individuals who are examining the incident or are participating on the RCA team with ideas, judgments, and proposed causes can create cognitive bias and groupthink. Cognitive biases can include “hindsight bias and outcome bias, two phenomena related to the tendency to perceive the results of prior decisions as more predictable than they actually were.”^35^ Groupthink occurs when the team tries to align the investigation's findings to the correctness of the expected outcome by prioritizing concurrence rather than voicing views that don't line up with those expectations.

### 7. Stand by your staff

Criminal investigation should be deterred, if possible, by the organization where the nurse is employed and invoked only in situations in which the nurse involved actually intended harm. Hospital legal departments need to proactively educate and develop a shared understanding about just culture and human error with district attorneys and malpractice lawyers in their communities. Representing or openly supporting their clinicians in court, if they're held individually accountable for systemic error, is critical. Organizational policies need to be explicit regarding self-reporting expectations for individuals and specify unambiguous actions of the organization should a staff member be prosecuted outside the confines of the organization's jurisdiction. Many statements calling out the condemnation of criminal prosecution for human error were provided by hospitals and professional organizations following the indictment and conviction of RaDonda Vaught, but most were vague restatements and self-serving. Caregivers need to know that they'll be unconditionally supported if they make a clear and honest human error or risky decision due to systemic circumstances.

### 8. Applaud, reward, and console

Finally, nurses' efforts in recognizing and reporting errors need to be expected, rewarded, and valued. Leaders need to implement visible and meaningful improvements to correct underlying systemic causes. To nurses, these are the rewards of reporting. When an outcome that isn't expected occurs, the intial response of support and consolation from leaders can mitigate the effects of a devestating failure. Guilt, self-blame, stress, and anxiety are second victim symptoms that, when addressed quickly, can be ameliorated.

## Moving forward

The acknowledgment that a large percentage of healthcare workers perceive that organizational response to error is punitive shouldn't come as a surprise. The exclusive use of RCA to investigate medical errors with little validation that this method of analysis produces changes that result in sustained improvement is an area ripe for study. Organizations need to incorporate systemic transformation, improve inadequate staffing, create decision tree policies and procedures, avoid second victimization, reduce preoccupation with risk management, and provide reality-oriented interprofessional education. A just culture should include open and honest sharing of the occurrence, supporting those involved, offering an apology, agreeing to remediate and correct to prevent further injury, and providing compensation when warranted.^32^ Nurse leaders need to use their courage to stand up with their direct care staff to call out injustices, inequities, and hazards. Organization executives need to support staff, through actions not statements, when threats to safety culture are present. Nurses and other healthcare workers should champion legislation that will protect healthcare workers from criminal charges for medical errors. When patient safety culture supports learning in unison from near-misses and actual events, then reliability and safety will improve. That's when patients can trust that their care is being safely administered, and they can embrace their difficult job of recovery without worry.

## INSTRUCTIONS Rebuilding trust in just culture

### TEST INSTRUCTIONS

- Read the article. The test for this nursing continuing professional development (NCPD) activity is to be taken online at www.NursingCenter.com/CE.
- You'll need to create an account (it's free!) and log in to access My Planner before taking online tests. Your planner will keep track of all your Lippincott Professional Development online NCPD activities for you.
- There's only one correct answer for each question. A passing score for this test is 7 correct answers. If you pass, you can print your certificate of earned contact hours and access the answer key. If you fail, you have the option of taking the test again at no additional cost.
- For questions, contact Lippincott Professional Development: 1-800-787-8985.
- Registration deadline is **September 5, 2025**.

### PROVIDER ACCREDITATION

Lippincott Professional Development will award 2.0 contact hours for this nursing continuing professional development activity.

Lippincott Professional Development is accredited as a provider of nursing continuing professional development by the American Nurses Credentialing Center's Commission on Accreditation.

This activity is also provider approved by the California Board of Registered Nursing, Provider Number CEP 11749 for 2.0 contact hours. Lippincott Professional Development is also an approved provider of continuing nursing education by the District of Columbia, Georgia,? West Virginia, New Mexico, South Carolina, and Florida, CE Broker #50-1223. Your certificate is valid in all states.

Payment: The registration fee for this test is $21.95.
